# Leveraging the Positive Deviance Approach to Drive Behavior Change in Noncommunicable Diseases: A Scoping Review

**DOI:** 10.1002/puh2.70074

**Published:** 2026-03-19

**Authors:** Oumnia Bouaddi, Imad El badisy, Hafida Charaka, Houda E. L. Kirat, Mouna Boucham, Majdouline Obtel, Asmae Khattabi, Kenza Hassouni, Lahcen Belyamani, Mohamed Khalis

**Affiliations:** ^1^ Department of Public Health and Clinical Research Mohammed VI Center for Research and Innovation (CM6RI) Rabat Morocco; ^2^ Mohammed VI International School of Public Health Mohammed VI University of Sciences and Health Casablanca Morocco; ^3^ Knowledge for Health Policy Center Mohammed VI University of Sciences and Health Casablanca Morocco; ^4^ Higher Institute of Nursing Professions and Technical Health Rabat Morocco; ^5^ Faculty of Medicine and Pharmacy of Rabat Rabat Morocco

**Keywords:** behavior change, chronic diseases, health promotion, healthy lifestyle, noncommunicable diseases, positive deviance

## Abstract

The positive deviance approach (PDA) to behavior and social change has been used to tackle child malnutrition in low‐resource settings and has yielded positive health outcomes. Yet, not much is known about its application in noncommunicable disease (NCD) prevention and promotion. We did a scoping review by searching three electronic databases; PubMed (MEDLINE), Scopus (ScienceDirect), and Google Scholar and gray literature websites for primary research studies published in any language, country, and on any date, reporting on the application of the positive deviance (PD) approach to NCDs. A total of 2802 records were retrieved and 26 articles were included in the final analysis. The majority of studies were related to the application of the PDA on physical activity, obesity, weight‐loss, and healthy eating, and the majority did not cover all steps of the PD approach and focused mainly on the identification of positive deviants and the identification of underlying PD behaviors and strategies, using a variety of quantitative and qualitative methods. All studies where PD behavior strategies were disseminated to nonpositively deviant communities and organizations yielded positive outcomes, such as clinically significant weight loss, and increased blood pressure control. The results of these studies suggest that positive deviants exhibiting exceptional performance exist in high‐risk NCD settings, and therefore researchers and public health practitioners in the field of NCD prevention and promotion can learn from their success.

## Graphical abstract

Positive deviants are individuals or groups who demonstrate unexpectedly positive outcomes in contexts where negative outcomes are the norm. In this study, positive deviants exhibited health‐promoting behaviors related to NCD risk factors. The use of these strategies led to positive health outcomes, which merit further exploration and integration into broader NCD prevention and health promotion initiatives.

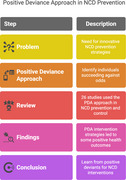

**Abbreviations**
NCDs: noncommunicable diseases
PD: positive deviance
PDA: positive deviance approach

## 1 Introduction

### 1.1 Definition and Application of the Positive Deviance Approach (PDA)

The PDA is a behavior and social change approach which posits that within a community, uncommon and healthy behaviors are already practiced by individuals known as positive deviants [1]. By identifying these outliers and working with them to promote positive health behaviors within their community, this approach can bring about change by capitalizing on existing resources, particularly in low resources settings [1]. The term “positive deviance” was first coined by Zeitlin in her pioneering work in the 1990s describing positive deviance in child nutrition [2]. In the 1990s, this approach was implemented through “Hearth” sessions to provide nutrition education to mothers of malnourished children in a community setting [3]. Hearth sessions are a series of 12‐day activities whereby children and their caregivers gather in home settings where volunteers—positively deviant mothers—prepare calorie‐dense and rich meal to feed the malnourished children using available resources [4].

The first application of the PDA took place in Vietnam as part of Jerry Sternin's work child on malnutrition with Peace Corps. By the end of a 2‐year pilot project, an 85% decrease in malnutrition was reported and children in the intervention group experiences better outcomes in terms of food portion, breastfeeding, growth, and the occurrence of respiratory infections. In addition, mothers in the intervention groups were more likely to engage in knowledge sharing about child nutrition with their peers [5, 6, 7]. Owing to the positive outcomes it yielded, the PDA has been extensively used to inform nutrition programs in more than 40 countries by USAID, World Vision, Mercy Corps, Save the Children, CARE, Plan International, Peace Corps, Food for the Hungry, and other agencies and organizations [8, 9, 10].

In 2003, the Nutrition Working Group in the CORE group organization developed a Positive Deviance/Hearth manual to guide program managers in the application of this approach to the design of sustainable rehabilitation programs for malnourished children [4]. Although the PDA is predominately used in the child nutrition, it has been applied to several other contexts. It has been deployed to promote condom use among sex workers in Georgia and Indonesia, to improve family planning methods in Guatemala, and to improve pregnancy outcomes in Egypt [1, 11]. It has also been used in efforts to improve education in the United States, to identify factors influencing sexual practices in West Africa, and to combat female circumcision in Egypt [11, 12, 13]. In addition, this approach has been widely applied to improve contraceptive practices [14], breastfeeding [12], HIV/AIDS prevention, improving adolescent sexual health [15], maternal and neonatal health [15], and hospital‐based infection prevention and control, such as MRSA prevention [16].

### 1.2 The Burden of Noncommunicable Diseases (NCDs) and the Rationale for PDA as a Preventive Approach

NCDs (including cardiovascular disease, cancer, chronic respiratory disease, and diabetes [17]) are the major cause of global mortality and the main cause of death and morbidity in the majority of low‐ and middle‐income countries (LMICs)—77% of deaths in LMICs are attributed to NCDs [18, 19]. Similarly, the burden of mental health issues has increased with an estimated 970 million people globally affected and anxiety being the most common disorder [20]. Common mental health disorders have been shown to coexist with NCDs with which they share many risk factors [18]. Due to their socioeconomic impact, NCDs are recognized as a major challenge to health and global development. The World Health Organization's Implementation Roadmap 2023–2030 to accelerate progress on preventing and controlling NCDs has laid out specific targets for member states and has highlighted risk factor management and behavior change that are central aspects in the prevention and management of NCDs [21]. As such, the most effective public health interventions stem from an in‐depth understanding of health behaviors and the circumstances in which they take place.

When externally designed and donor‐driven solutions are applied to local‐problem solving, their effectiveness is often conditioned by contextual factors. The characteristics of the PDA, such as its foundation on collective intelligence, cultural acceptability, and appropriateness, may indicate that it presents an alternative paradigm to traditional health promotion strategies and could be more sustainable. Although applications of this approach are common in several fields, its use in behavior change related to NCD prevention and control remains limited. A few pilot interventions have been tested and implemented regarding hypertension control [22], smoking cessation [23], diabetes [24], and mental health [25]. These interventions suggest that this approach may yield a positive impact on the adoption of healthy behaviors, particularly in low‐resource settings and within vulnerable communities. To date, no review has explored the use of this approach to promote behavior change in relation to NCDs. This scoping reviews aims to explore the application of the PDA in NCD prevention and control with a view to understanding its impact on behavior change of individuals, communities, and organizations.

## 2 Methods

We adopted the six‐stage scoping review methodology defined by Arksey and O'Malley, which is as follows: Identify the research question, identify relevant literature, select studies, chart the data, summarize, synthesize, report the results, and conduct expert consultation [18].

### 2.1 Identifying the Research Questions

This scoping review aims to provide an answer to the following questions: How has the PDA been used to understand and change NCD risk behaviors? What is the documented short, medium, and long‐term impact of these interventions?

### 2.2 Identifying Relevant Studies and Study Selection

In order to identify relevant studies, we searched three electronic databases; PubMed (MEDLINE), Scopus (ScienceDirect), and Google Scholar. Eligibility criteria included all primary research studies (quantitative, qualitative, and mixed‐methods) reporting on the use of the PDA in relation to NCDs (cardiovascular disease, cancer, chronic respiratory disease, diabetes, and mental health disorders) and risk factors (tobacco use, physical inactivity, unhealthy diet, and the harmful use of alcohol). Thus, we included studies reporting on any interventions related to promoting healthy diets, regular physical activity, avoiding smoking and second‐hand smoke, maintaining a healthy weight, and controlling blood pressure (BP), cholesterol, and blood sugar levels. We considered all intervention settings, including those implemented in healthcare or community settings, aimed at improving self‐management or the provision of preventive and control care for NCDs (e.g., counseling by healthcare providers). Letters to the editor and commentaries were excluded from this review. No language, date, or geographic restrictions were applied to the search. The search strategy was formulated using the PICO framework (Table 1) and combined terms for “NCDs” and “Positive Deviance” separated by Boolean operators. For Google Scholar, we used the advanced search function with keywords related to positive deviance, specific risk factors (e.g., diet, alcohol), and the four NCDs or mental health, separated by Boolean operators. The search strategy was refined and validated by all authors and performed by two authors (O.B. and I.B.). The final search strategy used for each database has been added to Table S1. In addition to peer‐reviewed literature, we performed forward and backward search and hand searched gray literature sites (The Positive Deviance Collaborative, UNICEF, and World Vision). The decision to include gray literature was based on this approach being widely used in projects by some international organizations. The retrieved records were imported into Mendeley reference manager where duplicates were removed. Title and abstract screening and full‐text screening was conducted independently by two reviewers. Any discrepancies at this stage were resolved by consensus with involvement of a third reviewer where necessary.

**TABLE 1:** PICO elements.

PICO	Eligibility criteria
Population	Individual patients, groups, or organizations
Intervention	Positive deviance related to NCD prevention and/or control
Comparison	Nonpositive deviant individuals or groups (where available)
Outcome	Any health‐related outcome/behavior

Abbreviation: NCD, noncommunicable disease.

### 2.3 Data Extraction

Two reviewers independently extracted the following data using a predefined form that was piloted and refined: information about the studies (location, year of study, study design, and study population), type of NCD and/or risk factor, stage of application of the PDA, description of the intervention, and documented outcomes (if any). All discrepancies at this stage were resolved by consensus with involvement of a third reviewer where necessary.

### 2.4 Data Synthesis and Reporting

**FIGURE 1:**
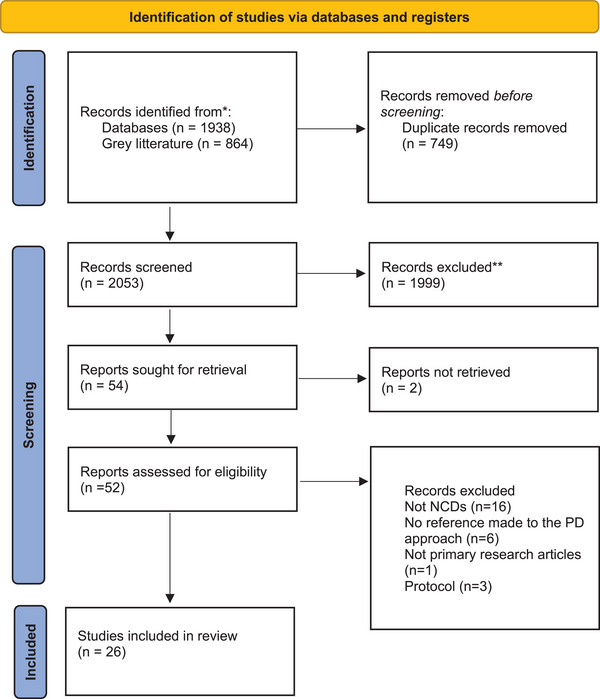
The positive deviance framework adapted from Refs. [1, 10].

A narrative summary of the findings and their implications was provided. The summary was guided by the positive deviance framework initially designed by Marsh et al. [1] that was adapted for the purposes of this review (Figure 1). This review was reported using standards and guidelines for reporting on scoping review PRISMA ScR [26].

## 3 Results

**FIGURE 2:**
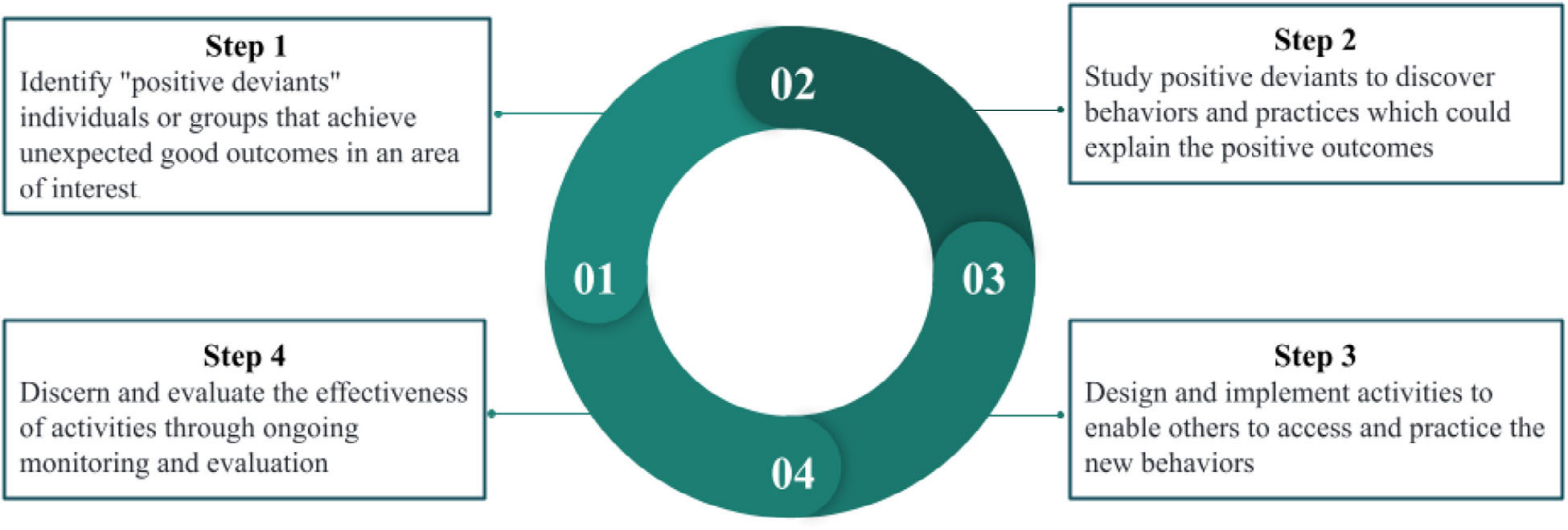
PRISMA flowchart.

A total of 1938 records were identified through database search and an additional 864 records were identified through Google Scholar and gray literature search. A total of 52 full‐texts were assessed for eligibility, and a total of 26 articles were included in the final analysis (Figure 2). The list of excluded articles with reasons has been added to Table S2. We present a narrative synthesis of the results below. A detailed data extraction form was added to Table S3.

### 3.1 Study Characteristics

Among included studies, 7 were qualitative, 10 were quantitative, 3 were trials, and 6 used a mixed‐methods approach. A total of 20 studies explored PDA in individuals and patients or communities, whereas a minority (6) explored positive deviance in providers (both organizations and health professionals). Regarding the NCDs and risk factors of interest, the majority of studies were related to physical activity, obesity, weight‐loss, and healthy eating (Table 2). In the following, we present the synthesis by grouping studies based on shared risk factors or diseases. Studies conducted in healthcare organization contexts are presented separately, rather than by disease areas (Table 2).

**TABLE 2:** General characteristics of included studies.

Characteristics	Number of studies
**Study design**	
Randomized controlled trials	3
Qualitative studies	7
Quantitative studies	10
Mixed‐methods studies	6
**Population of interest**	
Individual patients/communities	20
Providers/organizations	6
**NCD or risk factor of interest**	
Physical activity	3
Healthy eating	2
Obesity	7
Weight loss	5
Chronic respiratory diseases (COPD)	1
High blood pressure	1
Diabetes	3
Alcohol consumption	1
Cancer	1
Myocardial infarction	1

Abbreviation: NCD, noncommunicable disease.

### 3.2 Level of Application of the PDA

Table 3 shows the distribution of included studies across PDA framework steps. The identification of positive deviance (PD) individuals was done in all 26 studies, studying PD behavior was done in 20 studies. About six studies reported the design and/or implementation of intervention using positive deviance strategies [22, 27, 28, 29, 30, 31]. Of these, only four studies reported empirically assessed outcomes of the PDA interventions [22, 29, 32]. Positive deviants were identified in all studies using a variety of methods, including electronic health records (EHRs) and medical records [28, 33, 34], intercept interviews [35], on‐site observations [35], surveys, geospatial mapping (for healthcare organizations) [36], databases, and in‐depth interviews. Although the majority of studies used in‐depth interviews and focus‐group discussions in addition to quantitative methods, some studies used quantitative models to identify predictors and correlates of positively deviant outcomes.

**TABLE 3:** Positive deviance (PD) stages in the included studies.

First author	Step 1: Identifying positive deviants	Step 2: Studying the behaviors of positive deviants	Step 3: Designing and implementing activities	Step 4: Conducting impact evaluation
Abildso [34]	**˙**	**˙**		
Anderson [39]	**˙**	**˙**		
Curry [40]	**˙**	**˙**		
Davis [33]	**˙**			
Canavan [38]	**˙**	**˙**		
Fiechtner [28]	**˙**			**˙**
Foster [26]	**˙**		**˙**	**˙**
Foster [36]	**˙**	**˙**		
Gabbay [42]	**˙**	**˙**		
Hassim [47]	**˙**	**˙**		
Mateo [54]	**˙**	**˙**		
Breathett [48]	**˙**	**˙**		
Kinsey [49]	**˙**	**˙**		
Kraschnewski [31]	**˙**		**˙**	**˙**
Kraschnewski [50]	**˙**			
Banerjee [51]	**˙**	**˙**		
Bolen [21]	**˙**	**˙**	**˙**	**˙**
Sharifi [32]	**˙**	**˙**		
Spurr [37]	**˙**	**˙**		
Stuckey [29]	**˙**	**˙**		
Taliani [41]	**˙**	**˙**		
Taveras [27]	**˙**	**˙**	**˙**	
Topmiller [35]	**˙**	**˙**	**˙**	
Tucker [52]	**˙**	**˙**		
Vossenaar [30]	**˙**	**˙**	**˙**	
Wilson [53]	**˙**			

### 3.3 PDA Applied to Child Obesity and Healthy Eating

Several included studies deployed the positive deviant approach to identify parent–child pairs and analyze the behaviors leading up to positive outcomes in terms of child obesity and reduced BMI maintenance [27, 28, 29, 37]. Several positively deviant behaviors have been identified in parents of PD children such as making healthy snacks available for the child to access at will; lower juice and higher yogurt consumption; greater internalization of reasons for behavior change; greater parental recognition of emotional eating; avoiding purchasing unhealthy snacks altogether; greater organization and planning around meals and snacks and shared decision‐making in the family, setting up consistent rules for snacking, screen time and physical activity, being involved in decision‐making with their child's healthcare provider regarding weight management and use of available community resources to support change [27, 28, 29, 37]. In addition, peer‐relationship (e.g., not being bullied or teased, obtaining positive support from family), feeling good about oneself, fitting into age‐appropriate clothing, and being able to keep up with other children, while being physically active, were also identified as key motivating factors for behavior changes among positively deviant children [33]. Two studies described the design and implementation of PDA interventions to address healthy eating and weight loss among children. These included a parent peer mentor program, whereby parents of normal weight children in high‐risk Hispanic communities were trained as peer mentors to parents of obese children in the same community [27], and a Youth and Parent advisory board was set up to inform and support the design and implementation of the intervention that integrated PD strategies [28]. In both studies, the PDA approach yielded positive outcomes such as increased child‐self‐control, decreased use of food as a reward for the child, increased empowerment of the child to contribute to meal planning at home and increased parental control over their child's food intake [27], and clinically important weight loss [28]. These results were achieved even when comparing with other interventions such as health education and behavior change interventions by CHWs [27].

### 3.4 PDA Applied to Physical Activity

Among physically active adolescents, one study identified recreational time, increased sense of wellness, age, and family support as predictors of physical activity [38]. Among adults, successful positive deviants in terms of weight loss were identified as those who weigh themselves more often, monitor their food consumption, and physical activity [30]. In another study, a pre‐identified list of weight‐loss practices from positive deviant individuals was built in to a website—which prompted participants to develop a weight‐loss plan by selecting their preferred practices and setting weekly goals for practice [32]. This PDA activity led to a 1.4 kg mean weight loss in the intervention group compared to a mean weight gain of 0.6 kg in the control group [32]. In another study by Canavan et al., semi‐structured interviews with community leaders involved in health promotion initiatives and programs (healthy eating and active living lifestyles) were conducted to identify community‐level factors responsible for the lower‐than‐expected obesity rate [39]. The ability to foster connections and transfer ownership to communities was some community‐level factors underlying the PD outcomes in this study [39]. Similarly, Abildso et al. identified several community levels factors in physically active rural communities—positive outliers—such as favorable social norms, greater infrastructure and capital to support community activities, and greater human and organizational capital [35].

### 3.5 PDA Applied to Diabetes

Only one study reported on the use of the PDA in diabetes. This study compared US countiesthat reported higher percentages of diabetic Medicare beneficiaries receiving adequate care in positively deviant “bright counties,” compared to their counterparts that have similar demographic and socioeconomic characteristics, but poorer diabetes care outcomes [36]. The positive deviant counties had higher rates of PCPs [36]. Peer county groups linking the positive deviant counties to their counterparts with similar demographics and socioeconomic characteristics were created; however, this process has not been elucidated and the intervention has not been described [36].

### 3.6 PD Approach Applied to Healthcare Organizations

Five studies described the use of the PDA to examine the performance of healthcare organizations and providers and identify the processes leading to the observed performance [22, 40, 41, 42, 43]. In one study, health clinics that demonstrated superior BP control, compared to their counterparts in the same network of clinics, were identified, and interviews with clinic leaders identified accurate BP measurement and repeat measurement, timely follow‐up, prioritization of low‐cost once daily medication and adequate communication, and trusting relationships with patients as key factors behind the PD outcomes [22]. The practices were then disseminated through a series of conferences. The intervention reported a 7% increase in the proportion of patients with BP controlled (<140/90 mmHg) across 5‐year period [22]. In another study, semi‐structured interviews with providers were conducted to identify organizational factors and factors related to the practice environment, which underlie low chronic respiratory diseases (COPD) readmission rates in veteran health facilities [40]. This study reported higher relational and care coordination (e.g., efficient communication and collegial working environment), whereas their counterparts at high‐readmission sites reported important structural barriers such as poor communication and occasional antagonism between PCPs and specialists [40]. In another study by Gabbay et al. on diabetic medical homes, interviews with leaders showed that the positive deviant high performing practices in terms of morbidity and mortality had more structural resources such as EHR [42]. In addition, significant differences between the high and low performing comparison groups in leadership style and key processes (e.g., monitoring and evaluation) and team dynamics, were identified [43].

## 4 Discussion

The aim of this scoping review was to explore the use of the PDA in the prevention and control of NCDs and their risk factors at individual, community and healthcare organization levels. The majority of studies in this review aimed to identify positively deviant individuals, communities, and organizations and assess the salient behaviors and factors leading up to the unexpected positive health outcomes. Only a minority of studies documented the impact of interventions that integrate the pre‐identified PD strategies [22, 29, 32]. These studies indicate that the PD approach may be a viable one to behavior changes and is able to produce outcomes similar or superior to community‐health worker models in health promotion, and superior outcomes when compared to no interventions [27, 32].

The majority of studies focused on the identification of positive deviants (Step 1) and the identification of underlying PD behaviors and strategies, using a variety of quantitative and qualitative methods. In most studies focusing on the first two steps, positive deviants were adequately defined and cut‐off points (e.g., BMI) were justified. However, no standard definition of PD has been observed across the studies, which is consistent with the literature exploring the PD methodology [44]. The third and fourth steps in the application of the PD approach were rarely conducted suggesting the novelty of the application of this approach in NCD control and prevention. Among studies that conducted the fourth stage of the PD process, only two studies had an adequate follow‐up period: 5 and 1 years, respectively [22, 27]. In addition, although PD strategies were integrated into intervention studies, positive deviants were only involved in the implementation process in one study [27, 28]. In contrast, the findings of an included study emphasized the role of community ownership in the achievement of unexpected positive outcomes in PD communities [39]. This suggests that weaving participatory approaches in PD interventions may increase the likelihood of their success. In fact, previous applications showed a wider acceptance of PD strategies among staff in healthcare organizations and increased engagement and ownership [16].

The PD approach has been extensively used in for quality improvement in healthcare organizations and to control hospital‐associated infections [16, 45]. Existing evidence strongly suggest that the PDA was beneficial in achieving sustained and lasting reductions in MRSA healthcare‐associated infections, through the deployment of positive deviants in empowerment and role modeling of the staff [16, 46]. The use of the PD approach to identify practices in high performing practices and healthcare organization presents considerable potential benefit, particularly when considering, the cost of long‐term NCD care and control. The bottom‐up nature of the approach also indicates that it may be useful in driving behavior change, because it presents a framework for identifying and scaling‐up home‐grown solutions and therefore might gather more acceptance.

The majority of studies adopted the PD approach to identify and analyze successful healthy eating and weight‐control practices. Regarding healthy eating, adherence to nutrient‐rich diet often comes at a high price, which poses a significant barrier to healthy eating particularly in resource‐limited settings. However, positive deviants could achieve similar outcomes in terms of healthy eating compared to nonpositive deviants, at a lower cost [34]. In most weight‐loss interventions, the behaviors identified through the PD approach were not different from others identified in other weight‐control interventions; however, the use of qualitative data on these practices may allow for the creation of sustainable interventions with limited oversight [32].

Despite previous successes of the PD approach in addressing malnutrition, hospital‐based infections, and reducing door‐to‐balloon time in acute myocardial infarction, the understanding of how the approach operates and the evidence supporting its effectiveness in other areas of application, including NCD control and prevention, remain limited. On the basis of the findings in this review, PD might be an alternative approach to traditional health promotion models given the positive reported outcomes in studies where it was applied [22, 28, 29, 32]. However, analyses comparing the cost‐effectiveness of this approach to that of other interventions (e.g., deployment community‐healthy workers) are needed and constitute an appropriate target for future research. In addition, the initial application of PD in community‐based malnutrition interventions is well‐documented and straightforward [47]. Transferring this know‐how to NCD prevent and control may be challenging given the highly interconnected nature of the diseases and risk factors. Nevertheless, the main feature of the PD factor remains the leveraging of existing resources to overcome behavioral challenges and promote behavior change maintenance. Given that the burden of NCDs is disproportionately born by those in limited‐resource settings, the PD approach has the potential to create sustainable solutions.

### 4.1 Strengths and Limitations

This review has several strengths. First, this is the first review to explore the application of the PD approach in the field of NCD prevention and control. Second, the research question was identified with input from Ministry of Health focal points in the Department of NCDs in Morocco and therefore aligns with the interests of key decision makers in the country. In addition, this review was conducted by a multidisciplinary team with diverse experience in public health, epidemiology, and social and behavioral sciences. This review also has some limitations. Despite the inclusivity of the search strategies used, we may not have been able to identify studies that did not explicitly state the use of the PD approach. Furthermore, excluding languages other than English and French may have biased our results; however, given the novelty of the approach in this area of application, we believe that to be unlikely. Finally, quality appraisal of the included study was not conducted.

## 5 Conclusions

Positive deviants are individuals and groups that display unexpected positive outcomes rather than expected negative outcomes. This review shows that positive deviants exist and have been identified in different settings, and their behaviors have been analyzed to identify salient factors that help them adopt protective and healthy behaviors. Few studies have integrated PD strategies into interventions. The results of these studies are promising and suggest that positive deviants exhibiting exceptional performance exist in high‐risk settings and therefore researchers and public health planners can learn from their success.

## Author Contributions

Oumnia Bouaddi was involved in study design, development of scoping review search, data collection, data analysis, data interpretation, figures, tables, and writing. Imad Elbadisy was involved in study design, scoping review search, abstract and full‐text screening, data extraction, and data analysis. Hafida Charaka was involved in study design, scoping review search, abstract and full‐text screening, data extraction, and data analysis. Houda E. L. Kirat was involved in study design, scoping review search, abstract and full‐text screening, data extraction, and data analysis. Mouna Boucham was involved in study design, scoping review search, abstract and full‐text screening, data extraction, and data analysis. Kenza Hassouni was involved in study design scoping review search, abstract and full‐text screening, data extraction, and data analysis. Majdouline Obtel was involved in the revision of the manuscript and study supervision. Lahcen Belyamani was involved in the revision of the manuscript and study supervision. Asmae Khattabi was involved in the revision of the manuscript and study supervision. Mohamed Khalis was involved with the development of the scoping review search, data verification, data interpretation, revisions to manuscript, and study supervision.

## Ethics Statement

The authors have nothing to report.

## Conflicts of Interest

The authors declare no conflicts of interest.

## Supporting information




**Supporting File:** puh270074‐sup‐0001‐SuppMat.docx

## Data Availability

The data that support the findings of this study are available in the Supporting Information section of this article.

## References

[1] D. R. Marsh , D. G. Schroeder , K. A. Dearden , J. Sternin , and M. Sternin , “The Power of Positive Deviance,” British Medical Journal 329 (2004): 1177–1179. 15539680 10.1136/bmj.329.7475.1177 PMC527707

[2] M. F. Zeitlin , H. Ghassemi , and M. Mansour , United Nations University & Joint WHO/UNICEF Nutrition Support Programme. Positive deviance in child nutrition: with emphasis on psychosocial and behavioural aspects and implications for development. United Nations University Press. ((1990), https://iris.who.int/handle/10665/39406.

[3] O. Wollinka , E. Keeley , B. Burkhalter , and N. Bashir , Hearth Nutrition Model: Applications in Haiti Vietnam, and Bangladesh. US Agency for International Development (1997).

[4] CORE Group , Positive Deviance/Hearth: A Ressource Guide for Sustainably Rehabilitating Malnourished Children (CORE Group, 2003).

[5] T. Sripaipan , D. G. Schroeder , D. R. Marsh , et al., “Effect of an Integrated Nutrition Program on Child Morbidity Due to Respiratory Infection and Diarrhea in Northern Viet Nam,” Sage Journals 23 (2018): 70–77, 10.1177/15648265020234S110. 12503234

[6] D. R. Marsh , H. Pachón , D. G. Schroeder , et al., “Design of a Prospective, Randomized Evaluation of an Integrated Nutrition Program in Rural Viet Nam,” Food and Nutrition Bulletin 23 (2002): 36–47, 10.1177/15648265020234s206. 12503230

[7] J. L. Hendrickson , K. Dearden , H. Pachón , N. H. An , D. G. Schroeder , and D. R. Marsh , “Empowerment in Rural Viet Nam: Exploring Changes in Mothers and Health Volunteers in the Context of an Integrated Nutrition Project,” Sage Journals 23 (2016): 86–94, 10.1177/15648265020234S212. 12503236

[8] U. A. T. Mackintosh , D. R. Marsh , and D. G. Schroeder , “Sustained Positive Deviant Child Care Practices and Their Effects on Child Growth in Viet Nam,” Food and Nutrition Bulletin 23, no. 4_suppl_1 (2002): 16–25, 10.1177/15648265020234S104. 12503228

[9] Peace Corps , Hearth Nutrition Guide (Peace Corps, 2008), https://files.peacecorps.gov/multimedia/pdf/media/PCTimes2007_11.pdf.

[10] Nutrition Working Group, CORE Group . (June 2005). Positive Deviance/Hearth Essential Elements: A Resource Guide for Sustainably Rehabilitating Malnourished Children (Addendum). Washington, DC: CORE Group.

[11] Positive Deviance Collaborative , All Projects (Positive Deviance Collaborative, n.d.), http://positivedeviance.org/all‐projects.

[12] G. Ara , M. Khanam , N. Papri , et al., “Peer Counselling Improves Breastfeeding Practices: A Cluster Randomized Controlled Trial in Urban Bangladesh,” Maternal & Child Nutrition 14 (2018): e12605, 10.1111/MCN.12605. 29660858 PMC6055706

[13] S. Babalola , D. Awasum , and B. Quenum‐Renaud , “The Correlates of Safe Sex Practices Among Rwandan Youth: A Positive Deviance Approach,” African Journal of AIDS Research 1 (2002): 11–21, 10.2989/16085906.2002.9626540. 25871705

[14] H. Kosugi , A. Shibanuma , J. Kiriya , et al., “Positive Deviance for Promoting Dual‐Method Contraceptive Use Among Women in Uganda: A Cluster Randomised Controlled Trial,” BMJ Open 11 (2021): e046536, 10.1136/BMJOPEN-2020-046536. PMC8375745 34408034

[15] D. R. Marsh , M. Sternin , R. Khadduri , et al., “Identification of Model Newborn Care Practices Through a Positive Deviance Inquiry to Guide Behavior‐Change Interventions in Haripur, Pakistan,” Food and Nutrition Bulletin 23, no. 4_suppl_1 (2002): 107–116, 10.1177/15648265020234S115. 12503239

[16] N. M. O. Escobar , I. A. V. Márquez , J. A. Quiroga , et al., “Using Positive Deviance in the Prevention and Control of MRSA Infections in a Colombian Hospital: A Time‐Series Analysis,” Epidemiology and Infection 145 (2017): 981–989, 10.1017/S095026881600306X. 28065202 PMC9507818

[17] World Health Organization , Noncommunicable Diseases , https://www.who.int/news‐room/fact‐sheets/detail/noncommunicable‐diseases.

[18] D. J. Stein , C. Benjet , O. Gureje , et al., “Integrating Mental Health With Other Non‐Communicable Diseases,” Bmj 364 (2019): l295, 10.1136/BMJ.L295. 30692081 PMC6348425

[19] World Health Organization , Mental Health , https://www.who.int/health‐topics/mental‐health#tab=tab_2.

[20] World Health Organization , Implementation Roadmap 2023–2030 for the Global Action Plan , https://www.who.int/teams/noncommunicable‐diseases/governance/roadmap.

[21] S. D. Bolen , T. E. Love , D. Einstadter , et al., “Improving Regional Blood Pressure Control: A Positive Deviance Tiered Intensity Approach,” Journal of General Internal Medicine 36 (2021): 1591–1597, 10.1007/S11606-020-06480-Z. 33501526 PMC8175516

[22] N. Awofeso , N. Awofeso , T. Irwin , and G. Forrest , “Using Positive Deviance Techniques to Improve Smoking Cessation Outcomes in New South Wales Prison Settings,” Health Promotion Journal of Australia 19 (2008): 72–73. 18481937

[23] F. S. Pather and W. D. Tucker , A Health Informatics Model for User‐Centred Design Using a Positive Deviance Approach: A Case for Diabetes Self‐Management [PDF]. University of the Western Cape. (2020), https://uwcscholar.uwc.ac.za:8443/server/api/core/bitstreams/4a8c163f-f92d-4554-a3cb-4eadc4a96a1a/content.

[24] M. Bouman and A. Singhal , What Explains Enhanced Psychological Resilience of Students at VMBO Schools in the Netherlands? The Positive Deviance Approach in Action (Center for Media & Health, n.d.). https://www.media-gezondheid.nl/beheer/data/cmg.desh26.nl/uploads/Publicaties_en_downloads/PD_Approach_the_Netherlands_CMH_040914_fin.pdf.

[25] A. C. Tricco , E. Lillie , W. Zarin , et al., “PRISMA Extension for Scoping Reviews (PRISMA‐ScR): Checklist and Explanation,” Annals of Internal Medicine 169 (2018): 467–473, 10.7326/M18-0850/SUPPL_FILE/M18-0850_SUPPLEMENT.PDF. 30178033

[26] B. A. Foster , C. A. Aquino , M. Gil , J. A. L. Gelfond , and D. E. Hale , “A Pilot Study of Parent Mentors for Early Childhood Obesity,” Journal of Obesity 2016 (2016): 2609504, 10.1155/2016/2609504. 27379182 PMC4917692

[27] E. M. Taveras , R. Marshall , M. Sharifi , et al., “Connect for Health: Design of a Clinical‐Community Childhood Obesity Intervention Testing Best Practices of Positive Outliers,” Contemporary Clinical Trials 45 (2015): 287–295, 10.1016/J.CCT.2015.09.022. 26427562 PMC4753774

[28] L. Fiechtner , I. Castro , E. R. Cheng , et al., “Characteristics of Achieving Clinically Important Weight Loss in Two Paediatric Weight Management Interventions,” Pediatric Obesity 16 (2021): e12784, 10.1111/IJPO.12784. 33734583 PMC8355061

[29] H. L. Stuckey , J. Boan , J. L. Kraschnewski , M. Miller‐Day , E. B. Lehman , and C. N. Sciamanna , “Using Positive Deviance for Determining Successful Weight‐Control Practices,” Qualitative Health Research 21 (2011): 563–579, 10.1177/1049732310386623. 20956609 PMC3612888

[30] M. Vossenaar , E. Mayorga , M. J. Soto‐Méndez , et al., “The Positive Deviance Approach Can be Used to Create Culturally Appropriate Eating Guides Compatible With Reduced Cancer Risk,” Journal of Nutrition 139 (2009): 755–762, 10.3945/jn.108.100362. 19225133

[31] J. L. Kraschnewski , H. L. Stuckey , L. S. Rovniak , et al., “Efficacy of a Weight‐Loss Website Based on Positive Deviance. A Randomized Trial,” American Journal of Preventive Medicine 41 (2011): 610–614, 10.1016/J.AMEPRE.2011.08.012. 22099238 PMC12456025

[32] M. Sharifi , G. Marshall , R. Goldman , et al., “Exploring Innovative Approaches and Patient‐Centered Outcomes From Positive Outliers in Childhood Obesity,” Academic Pediatrics 14 (2014): 646–655, 10.1016/J.ACAP.2014.08.001. 25439163 PMC4322896

[33] C. Davis , A. Drewnowski , and A. Aggarwal , “Eating Well and Paying Less: A Positive Deviance Study” (Thesis, University of Washington, 2014), https://digital.lib.washington.edu/server/api/core/bitstreams/953257c2-029b-40a8-b6a3-6f52a85b05cd/content.

[34] C. G. Abildso , C. K. Perry , L. Jacobs , M. Renée Umstattd Meyer , M. McClendon , and M. B. Edwards , “What Sets Physically Active Rural Communities Apart From Less Active Ones? A Comparative Case Study of Three US Counties,” International Journal of Environmental Research and Public Health 18 (2021): 10574, 10.3390/IJERPH182010574. 34682319 PMC8535724

[35] M. Topmiller , A. M. Kieber‐Emmons , K. Shaak , and J. L. McCann , “Identifying Bright Spot Counties for Appropriate Diabetes Preventive Care: A Geospatial, Positive Deviance Approach,” Journal of Primary Prevention 41 (2020): 431–443, 10.1007/S10935-020-00601-4. 32642939

[36] B. A. Foster , J. Farragher , P. Parker , and D. E. Hale , “A Positive Deviance Approach to Early Childhood Obesity: Cross‐Sectional Characterization of Positive Outliers,” Childhood Obesity 11 (2015): 281–288, 10.1089/CHI.2014.0098. 25885174 PMC4484711

[37] S. Spurr , J. Bally , and K. Trinder , “Predictors of Physical Activity in Positive Deviant Adolescents,” Journal of Pediatric Nursing 31 (2016): 311–318. 26725700 10.1016/j.pedn.2015.11.006

[38] M. E. Canavan , E. Cherlin , S. Boegeman , E. H. Bradley , and K. M. T. Slagle , “Community Factors Related to Healthy Eating & Active Living in Counties With Lower Than Expected Adult Obesity Rates,” BMC Obesity 3 (2016): 1–9, 10.1186/S40608-016-0129-X/TABLES/2. 27891242 PMC5114811

[39] E. Anderson , R. S. Wiener , K. Resnick , A. R. Elwy , and S. T. Rinne , “Care Coordination for Veterans With COPD: A Positive Deviance Study,” American Journal of Managed Care 26 (2020): 63–68, 10.37765/AJMC.2020.42394. 32059093 PMC7853407

[40] L. A. Curry , E. Spatz , E. Cherlin , et al., “What Distinguishes Top‐Performing Hospitals in Acute Myocardial Infarction Mortality Rates? A Qualitative Study,” Annals of Internal Medicine 154 (2011): 384–390, 10.7326/0003-4819-154-6-201103150-00003. 21403074 PMC4735872

[41] C. A. Taliani , P. L. Bricker , A. M. Adelman , P. F. Cronholm , and R. A. Gabbay , “Implementing Effective Care Management in the Patient‐Centered Medical Home,” American Journal of Managed Care 19 (2013): 957–964, http://www.ajmc.com/publications/issue/2013/2013-1-vol19-n12/implementing-effective-care-management-in-the-patient-centered-medical-home. 24512033

[42] R. A. Gabbay , M. W. Friedberg , M. Miller‐Day , P. F. Cronholm , A. Adelman , and E. C. Schneider , “A Positive Deviance Approach to Understanding Key Features to Improving Diabetes Care in the Medical Home,” Annals of Family Medicine 11 (2013): S99–S107, 10.1370/AFM.1473. 23690393 PMC3707253

[43] B. A. Foster , K. Seeley , M. Davis , and J. Boone‐Heinonen , “Positive Deviance in Health and Medical Research on Individual Level Outcomes—A Review of Methodology,” Annals of Epidemiology 69 (2022): 48–56, 10.1016/J.ANNEPIDEM.2021.12.001. 34915122 PMC9081135

[44] R. Baxter , N. Taylor , I. Kellar , and R. Lawton , “What Methods Are Used to Apply Positive Deviance Within Healthcare Organisations? A Systematic Review,” BMJ Quality & Safety 25 (2016): 190–201, 10.1136/BMJQS-2015-004386. PMC4789698 26590198

[45] M. A. Alzunitan , M. B. Edmond , M. A. Alsuhaibani , R. J. Samuelson , M. L. Schweizer , and A. R. Marra , “Positive Deviance in Infection Prevention and Control: A Systematic Literature Review,” Infection Control and Hospital Epidemiology 43 (2022): 358–365, 10.1017/ICE.2020.1256. 33172508

[46] A. Chakraborty and H. Siriwardane , “Identification of Positive Deviance—Methodology Development,” in Proceedings of the International Conference on Managing the Asian Century (Springer Science+Business Media Singapore, 2013), 421–428.

[47] N. Hassim , P. R. Fernandez , N. K. Hoong , et al., “Childhood Obesity: Health Communication Perspectives of Malaysian Parents During COVID‐19,” Search‐Journal of Media and Communication Research 13 (2021): 141–156.

[48] K. Breathett , L. N. Kohler , C. B. Eaton , et al., “When the at‐Risk Do Not Develop Heart Failure: Understanding Positive Deviance Among Postmenopausal African American and Hispanic Women,” Journal of Cardiac Failure 27 (2021): 217–223, 10.1016/J.CARDFAIL.2020.11.009. 33232822 PMC7880886

[49] A. W. Kinsey , M. L. Segar , D. J. Barr‐Anderson , M. C. Whitt‐Glover , and O. Affuso , “Positive Outliers Among African American Women and the Factors Associated With Long‐Term Physical Activity Maintenance,” Journal of Racial and Ethnic Health Disparities 6 (2019): 603–617. 30644068 10.1007/s40615-018-00559-4 PMC6500467

[50] J. L. Kraschnewski , C. N. Sciamanna , K. I. Pollak , H. L. Stuckey , and N. E. Sherwood , “The Epidemiology of Weight Counseling for Adults in the United States: A Case of Positive Deviance,” International Journal of Obesity 37 (2013): 751–753, 10.1038/IJO.2012.113. 22777541 PMC4429525

[51] E. S. Banerjee , S. J. Herring , K. Hurley , K. Puskarz , K. Yebernetsky , and M. LaNoue , “Determinants of Successful Weight Loss in Low‐Income African American Women: A Positive Deviance Analysis,” Journal of Primary Care and Community Health 9 (2018): 2150132718792136, 10.1177/2150132718792136. PMC6081755 30084705

[52] M. Tucker and G. E. Harris , “Alcohol Use Among University Students: Considering a Positive Deviance Approach,” Journal of Health Psychology 21 (2016): 1918–1927, 10.1177/1359105314568577. 25637069

[53] B. Wilson , C. L. Tseng , O. Soroka , L. M. Pogach , and D. C. Aron , “Identification of Outliers and Positive Deviants for Healthcare Improvement: Looking for High Performers in Hypoglycemia Safety in Patients With Diabetes,” BMC Health Services Research [Electronic Resource] 17 (2017): 1–10, 10.1186/S12913-017-2692-3/FIGURES/4. 29145834 PMC5691393

[54] K. F. Mateo , E. S. Banerjee , S. J. Herring , K. E. Hurley , et al., “What PCP‑Related Factors Contribute to Successful Weight Loss Among Positive Deviant Low‑Income African‑American Women?,” Journal of Clinical Outcomes Management 24 no. 5 (2017). May 8, 2017.

