# Correction to “Leveraging the Positive Deviance Approach to Drive Behavior Change in Noncommunicable Diseases: A Scoping Review”

**DOI:** 10.1002/puh2.70315

**Published:** 2026-07-23

Bouaddi O, El badisy I, Charaka H, et al. “Leveraging the Positive Deviance Approach to Drive Behavior Change in Noncommunicable Diseases: A Scoping Review,” Public Health Challenges. 2026;5. https://doi.org/10.1002/puh2.70074.

The author's name “Houda E. L. Kirat” was corrected to Houda El Kirat.

The affiliation of the author Imad El Badisy should AI and Data Science Service, Mohammed VI Center for Research and Innovation (CM6RI), Rabat, Morocco.

We apologize for these errors.

