# Supplementary material for: Leveraging the Positive Deviance Approach to Drive Behavior Change in Noncommunicable Diseases: A Scoping Review
Source: Public Health Chall. 2026 Mar 19;5(1):e70074. doi: 10.1002/puh2.70074 (PMC13098055; doi:10.1002/puh2.70074)
Supplement: Supplementary file 1 — Supporting File: puh270074‐sup‐0001‐SuppMat.docx [file PUH2-5-e70074-s001.docx]

**Table 1. Search strategies**

| **PUBMED** |
| --- |
| ("Positive deviance"[tiab] OR "Positive deviance approach"[tiab]) AND (Chronic illness [Mesh] OR Health behavior* [Mesh] OR "Non-communicable diseases" [Text Word] OR"Diabetes mellitus" [Mesh] OR "Cardiovascular Diseases"[Mesh] OR "Respiration Disorders"[Mesh] OR "Neoplasms" [Mesh] OR "Obesity" [Mesh] OR "Mental disorders"[Mesh] ) |
| ("Positive deviance"[tiab] OR "Positive deviance approach"[tiab]) AND ("Alcohol" [Text Word] OR "Alcohol Drinking" [Mesh] OR "Diet" [Text Word] OR "Diet" [Mesh] OR "Nutrition" [Text Word] OR "Exercise" [Text Word] OR "Exercise" [Mesh] OR "Feeding Behavior" [Mesh] OR "Smoking" [Text Word] OR "Tobacco Use" [Mesh] OR "Physical activity" [Text Word]) |
| ("Positive deviance"[tiab] OR "Positive deviance approach"[tiab]) AND ("Alcohol" [Text Word] OR "Alcohol Drinking" [Mesh] OR "Diet" [Text Word] OR "Diet" [Mesh] OR "Nutrition" [Text Word] OR "Exercise" [Text Word] OR "Exercise" [Mesh] OR "Feeding Behavior" [Mesh] OR "Smoking" [Text Word] OR "Tobacco Use" [Mesh] OR "Physical activity" [Text Word]) AND (Chronic illness [Mesh] OR Health behavior* [Mesh] OR "Non-communicable diseases" [Text Word] OR"Diabetes mellitus" [Mesh] OR "Cardiovascular Diseases"[Mesh] OR "Respiration Disorders"[Mesh] OR "Neoplasms" [Mesh] OR "Obesity" [Mesh] OR "Mental disorders"[Mesh]) |
| ("Positive deviance"[tiab] OR "Positive deviance approach"[tiab] or "Positive outliers"[tiab] OR "Positive deviance"[Text Word] OR "Positive deviance approach"[Text Word]) AND (Chronic illness [Mesh] OR Health behavior* [Mesh] OR "Non-communicable diseases" [Text Word] OR "Behavior change"[Text Word]) |
| ("Positive deviance"[tiab] OR "Positive deviance approach"[tiab] or "Positive outliers"[tiab] OR "Positive deviance"[Text Word] OR "Positive deviance approach"[Text Word]) AND ("Health Knowledge, Attitudes, Practice"[Mesh] OR "Health Promotion / methods*"[Mesh] OR "Health behavior*"[Mesh]) |
| (“positive deviance”[Title/Abstract]) OR (“positive deviance approach”[Title/Abstract])) OR (“positive outliers”[Title/Abstract]) |
| **SCOPUS** |
| ("Positive deviance" OR "Positive deviance approach" OR "positive outliers" OR "positive deviant") AND ("Alcohol" OR "Alcohol Drinking" OR "Diet" OR "Nutrition") |
| ("Positive deviance" OR "Positive deviance approach" OR "positive outliers") AND ( "Exercise" OR "Exercise" OR "Feeding Behavior" OR "Smoking" OR "Tobacco Use" OR "Physical activity" ) |
| ("Positive deviance" OR "Positive deviance approach" OR "positive outliers") AND ("Chronic illness" OR "Health behavior" OR "Non-communicable diseases" OR "cancer" OR "Diabetes" OR "Cardiovascular Disease") |
| ("Positive deviance" OR "Positive deviance approach" OR "positive outliers" OR "positive deviant") AND ("Health behavior" OR "Non-communicable diseases" OR "Respiratory diseases" OR "Obesity" OR "Mental disorders") |
| ("Positive deviance" OR "Positive deviance approach" OR "Positive outliers" OR "positive deviant") AND ("Chronic disease" OR "Health behavior" OR "Non-communicable "diseases" OR "Behavior change") |
| ("Positive deviance" OR "Positive deviance approach" OR "positive outliers" OR "positive deviant") AND ("Health behavior" OR "Non-communicable diseases" OR "chronic disease" OR "behavior" OR "risk factor") AND ("Chronic disease" OR "Non-communicable diseases") |

**Table 2. List of excluded articles with reasons**

| **N** | **Title** | **Reason** |
| --- | --- | --- |
| **1** | Positive deviance approaches to improving vaccination coverage rates within healthcare systems: A systematic review | not NCDs |
| **2** | Beyond the hospital infection control guidelines: A qualitative study using positive deviance to characterize gray areas and to achieve efficacy and clarity in the prevention of healthcare-associated infections 11 Medical and Health Sciences 1117 Public Health and Health Services | not NCDs |
| **3** | Environmental factors associated with physical activity in rural U.S. counties | no direct mention of PD |
| **4** | Using positive deviance techniques to improve smoking cessation outcomes in New South Wales prison settings | full text not found |
| **5** | How do Positive Deviants Overcome Health-Related Stigma? An Exploration of Development of Positive Deviance Among People With Stigmatized Health Conditions in Indonesia | not NCDs |
| **6** | A county-level cross-sectional analysis of positive deviance to assess multiple population health outcomes in Indiana | not NCDs |
| **7** | Evaluation of different food safety education interventions | not NCDs |
| **8** | A randomized clinical trial of the effects of parent mentors on early childhood obesity: Study design and baseline data | protocol |
| **10** | Applying the concept of positive deviance to public health data: A tool for reducing health disparities: Special features: Methods | not NCDs |
| **11** | The Nutrition Transition Among the Andean Kichwas of Ecuador | Not NCDs |
| **12** | Qualitative Exploration Of Behaviors Related To Positive Child Growth In An Urban Slum Of Mumbai | Not NCDs |
| **13** | Positive Deviance as a Framework for Understanding Motivations and Barriers to Exercise for University Students at Campus Recreation | not primary R |
| **14** | Plate half-empty or half-full? Dietary continuity and change among the Blackfeet | Not NCDs |
| **15** | Quantifying parent engagement in the randomized Fuel for Fun impact study identified design considerations and BMI relationships | Not NCDs |
| **16** | Growing Healthy Together: A Randomized Clinical Trial Using Parent Mentors for Early Childhood Obesity in Low-Income, Latino Families | Full-text not found |
| **17** | “Food is something everyone should participate in”: A positive deviance approach to understanding the use of a food and nutrition app in low-income, Latino homes | Not NCDs |
| **18** | Efficacy of text messaging and personal consultation by pharmacy students among adults with hypertension: Randomized controlled trial | No direct mention of PD approach |
| **19** | Pilot test of an educational intervention to improve self-management of diabetes in persons living with HIV | No direct mention of PD approach |
| **20** | Survival after Acute Myocardial Infarction (SAMI) study: The design and implementation of a positive deviance study | protocol |
| **21** | Positive Deviance for Dual-Method Promotion among Women in Uganda: A Qualitative Study | Not NCDs |
| **23** | Positive Deviance: A Non-Normative Approach to Health and Risk Messaging | Not NCDs |
| **22** | Assessing Potential Glycemic Overtreatment in Persons at Hypoglycemic Risk | No direct mention of PD approach |
| **23** | Determinants of better health: A cross-sectional assessment of positive deviants among women in West Bengal | Not NCDs |
| **24** | Chronic health conditions and internet behavioral interventions: A review of factors to enhance user engagement | Not PD |
| **25** | Survival after Acute Myocardial Infarction (SAMI) study: The design and implementation of a positive deviance study | Protocol |
| **26** | Developing health promotion interventions: a Multisource Method applied to weight loss among low-income postpartum women | Not PD |
| **27** | Use of positive-negative deviant analyses to improve programme targeting and services: Example from the TamilNadu integrated nutrition project | Not NCDs |
| **28** | The use of nutritional'positive deviants' to identify approaches for modification of dietary practices | Not NCDs |

**Table 3. Application of the Positive Deviance Approach in the included studies**

| **First author** | **Year** | **Step 1** | **Step 2** | **Step 3** | **Step 4** | **Main findings** |
| --- | --- | --- | --- | --- | --- | --- |
| **E Canavan M.** | 2016 | -Definition of PD threshold for obesity  -PD countries were in lowest quartile nationally and located in a state with the higher than the national average in obesity rates. | -Semi-structured interviews with community leaders involved in activities that promoted healthy eating and active living lifestyles |  |  | PD counties leveraged strengths in their communities to combat obesity and improve healthy living, built more partnerships, foster connections, and transfer ownership to community members. |
| **Foster B.** | 2016 |  |  | -Parents of normal weight children in high-risk Hispanic communities trained (PD) as peer mentors to parents of obese children in the same community. | -Parents-child dyads randomized to receive either parent mentor interventions/ education by experienced CHWs  -One year follow-up | -Decrease in their adiposity (BMI 𝑧-score) in both groups.  The diet and activity changes that were measured were consistent with this decrease in adiposity, and the plateauing of those diet and activity changes between the end of the intervention at six months and the twelve-month follow up is consistent with their weight stabilization. we observed an increase in parental |
| **Foster B.** | 2015 | Parent-child dyads with a normal weight child despite an adverse environment (high local prevalence of overweight and obesity and a low-socioeconomic background). | Interviews with PD and non-PD parent-child to assess children’s general health, eating and activity practices, weight perceptions. |  |  | Major PD strategies identified: making healthy snacks available for the child to access at will; lower juice and higher yogurt consumption; greater internalization of reasons for behavior change; greater parental recognition of emotional eating; avoiding purchasing unhealthy snacks altogether; greater organization and planning around meals and snacks |
| **Sharifi M.** | 2014 | Children with maintained reduced BMI in “hotspots” | FGD with PD parent-children to identify perceptions, successful strategies and preferences among families of children. |  |  | -Parents: PD practices were 1) making changes as a family rather than solely for the child; 2) implementing consistent rules around snacking, screen time and activity 3) being involved in the decision-making with health care provider about child's weight management 4) using more immediate outcomes of weight management to motivate change, 5) leveraging community resources to support behavior change.  -Children: PD strategies were 1) emphasis on the value of positive support from family and peers in motivating behavior change. 2) shared desired outcomes such as not being bullied or teased, feeling good about oneself, fitting into age-appropriate clothing, and being able to keep up with other children while being physical active. |
| **Fiechtner L.** | 2021 |  |  |  | Measurement of clinically important weight loss (CIWL) in two trials STAR and Connect for Health after a one-year follow-up period | Children participating in two childhood obesity randomized controlled trials 26% were able to achieve CIWL or a BMI z-score decrease of by ≥ 0.2 units. In adjusted analyses, these children were characterized by younger age, lower baseline BMI status, and lower consumption of sugary beverages |
| **Taveras E.** | 2015 | Identification of children with maintained reduced BMI through electronic health records (EHR) | -Focus groups with positive deviant parent-children’s pairs.  -Setting up of a youth and parent advisory board to support the implementation of PD intervention. | Control group: enhanced primary care (e.g. flagging of children with BMI≥ 85th percentile, clinical decision support tools for pediatric weight management, parent educational materials, a Neighborhood Resource Guide and monthly text messages)  Intervention group: enhanced primary care plus contextually tailored, individual health coaching (twice-weekly text messages and telephone or video contacts every other month) to support behavior change and linkage of families to neighborhood resources. |  | - |
| **Stuckey H.** | 2011 | PD were individuals successful with long-term weight control (maintained a weight loss of at least 30 pounds for at least 1 year) | In-depth examination of a participant’s behaviors, attitudes, and barriers through in-depth interviews. |  |  |  |
| **Kraschnewski J.** | 2011 |  |  | -Participants randomized to control/intervention group  -Intervention group: accessed AchieveTogether website at least once weekly, provided target body weight at the initial log-in, were matched to role models closest to them, reviewed their role models’ strategies for implementing 36 PD weight-loss practices, viewed videos of role models incorporating the practices, were prompted to build a weight-loss plan by selecting their preferred practices and setting weekly goals. | -Outcomes measured: Height, weight and BP, caloric intake, Impact of Weight on Quality of Life questionnaire.  -Process measures: the number of website log-ins (derived from server registrations); website satisfaction; and weight control practices used | Mean weight loss among intervention participants was 1.4 kg (95% CI 0.5-2.2), compared with a mean weight gain of 0.6 kg (95% CI 0.3, 1.4) in control participants (p0.01). |
| **Kinsey A.** | 2019 | PD were AA or black women, at least 18 years of age, and self-reported currently being physically active for more than 6 months, on average, 250 minutes of moderate PA per week (66.7% greater than nationally recommended levels) | 1) Examination of the personal characteristics, PA patterns and behavioral practices of positive outliers among AA women and 2) comparison of characteristics of those who maintain PA at recommended levels (with those who maintained low PA volumes (LOW, 6 months) |  |  | PD behavior associated with PA maintenance: regular routine (i.e., active in the mornings before starting their day and scheduling PA during the week), use of goal-setting and self-monitoring practices; self-regulation practices known to support behavior changes and maintenance |
| **Seaton E.** | 2018 | PD were individuals who intentionally lost at least 10% of their maximum weight (not due to bariatric surgery, a medical problem, or childbirth) and maintained this loss for at least 6 months. | Interviews conducted with PD women |  |  | Among PD: Increased opportunity affected their successful weight loss, more likely to be currently making diet changes, use of creative tricks to prolong dietary change. |
| **Topmiller M.** | 2020 | Identification of bright spot (PD) counties with higher percentages of Medicare beneficiaries receiving appropriate diabetes preventive care | Investigation and comparison of resources and behaviors in high and low performance counties | -Creation of peer county groupings for case comparison  -Linkage of bright spot counties with priority counties (non-PD), with similar demographic and socioeconomic characteristics |  | -Bright spot counties (PD): mostly rural, older populations, higher rates of primary care physicians, more physically active and had lower rates of food insecurity  -Priority counties (non-PD) had higher rates of obesity and less access to physical activity resources than PD counties. |
| **Spurr S.** | 2015 | Survey of adolescents in schools and identification of PD adolescents (adequate physical activity PA). | Analysis of PA predictors in PD adolescents using a multidimensional and wellness adolescent model. |  |  | Significant predictors of PA in PD were; for girls: recreational time, an increased sense of wellness, age, and family support. For boys, use of recreational time |
| **Hassim N.** | 2021 | Semi structured interviews conducted to identify PD parents regarding the control of obesity in their children. | Parents interviewed about their perceptions towards health communication, and how the ensure their children abide by healthy eating habits, and how they seek information on childhood obesity intervention during COVID-19 |  |  | -Parents from M40 and B40 communities had insufficient information on nutrition for childhood development and prioritize convenience over long-term effects due to their hectic routines and household income  -Parents from T20 communities are more efficient in managing and applying knowledge due to higher health literacy and communication within their social groups (PD) |
| **Katrina F.** | 2017 | PD were African-American women patients with EMR-confirmed weight loss of at least 10% of patient’s maximum weight between 2007–2012 and maintenance of this loss for at least 6 months. |  |  |  | Patients in the population at high risk for obesity may benefit from their PCPs drawing connections between obesity and weight-related medical conditions and enhancing intrinsic motivation for weight loss. |
| **Shari D.** | 2021 | Identification of clinics in the same health system demonstrated a high degree of improvement and achievement of BP control | Interviews with leaders of PD clinics to determine what processes responsible improvements in BP control | -Dissemination of the PD practices through series of conference  -Implementation of PD practices in high, medium and low levels of intensity based on performance of clinics with regards to BP control (low performing facilities received more coaching in implementation) | -De-identified EHR data submitted every 6 months from 2007 through 2013  -Patient information was collected regarding age, sex, race/ethnicity, insurance type, diabetes diagnosis, BP, body mass index, preferred language, and home address (used to estimate household income and education) | -PD strategies in high performing clinics: (1) accurate BP and repeat measurement (2) timely follow up (3) EHR-based registry outreach to contact patients whose last BP was elevated and who had no follow-up appointment scheduled (4) a treatment algorithm which prioritized once-daily, low-cost medications; (5) a communication curriculum focused on building trusting relationships with patients  -Proportion of patients with BP controlled (< 140/90 mmHg) increased overall from 67% in 2013 to 74% in 2017  -Absolute improvement in BP control was 7.6 percent (95% CI 6.0–9.1) for all patients |
| **Khadijah B.** | 2021 | African-American and Hispanic women with the highest risk, were isolated for assessment and followed for incident heart failure hospitalization. | Identification of psychosocial protective factors against heart failure hospitalization (HFH) in high-risk postmenopausal AA women |  |  | -High-risk African-American women optimism and social strain were modestly associated with risk of incident heart failure hospitalization  -High risk Hispanics: living alone was significantly associated with higher risk of incident heart failure hospitalization in unadjusted models  -No significant associations found in the fully adjusted analysis |
| **Anderson E.** | 2020 | Selection of sites in the lowest and highest readmission quartiles, respectively in terms of COPD readmission | Interviews with PCPs and other specialist to identify organizational factors, including relational coordination, at Veteran affairs sites with high versus low COPD readmission healthcare providers in both high admission and low readmission for COPD |  |  | In low readmission (PD) facilities; Providers at low-readmission facilities described organizational environments that foster collegial working relationships and efficient communication, their counterparts at high-readmission sites reported issues in these domains and referenced significant structural barriers to care coordination, the practice environment at low-readmission sites, to the extent that it was adequately described by provider accounts, had a higher degree of relational coordination than the practice environment at high-readmission sites |
| **Taliani C.** | 2013 | The 5 practices (diabetic medical homes) with highest improvement in measures of diabetes care were identified as PD, whereas the 5 practices with the least improvement in these measures were selected as a comparison group (non-PD). | Leaders of each practice were surveyed to assess the structural capabilities, performance feedback, systems for communicating with diabetic patients, use of patient registries and HER, presence of staff trained to assist patient self-management.  -Staff (clinicians, administrators) surveyed to determine whether higher-performing practices had stronger mechanisms to cope with change and exhibited lower levels of clinician and staff burnout |  |  | -PD practices had higher structural capabilities (eg, EHR) than non-PD practices at baseline.  -Interviews revealed considerable differences between the groups in leadership styles and shared vision; sense, use, and development of teams; processes for monitoring progress and obtaining feedback; and presence of technologic and financial distractions. |
| **Gabbay A.** | 2013 | Selection of 2 groups, women who had attended the Honduran Health Alliance (HHA) clinic a community-based education, community development and clinical reproductive health program (PD) and women who had never attended (non-PD). | FGD conducted with one with women who had attended the HHA clinic and women who had never attended (reasons for attending or non-attending the HHA clinic, experiences with screening at the HHA clinic, and barriers to attending the HHA clinic). |  |  | Self-love and social support were identified as two constructs women employed to overcome barriers to screening. |
| **Wilson B.** | 2017 | Assessment of performance of high performing healthcare facilities in terms of diabetes overtreatment, in an opposite measure, undertreatment |  |  |  | Consistent high performing facilities for overtreatment had higher rates of undertreatment |
| **Tucker M.** | 2016 | Participants were asked to report the number of drinks consumed and the number of hours spent consuming those drinks. | -Self-efficacy was measured using Drinking Refusal Self-Efficacy Questionnaire–Revised Edition. Family contextual factors and personal commitment were assessed. -Multinomial regression was conducted. |  |  | Higher personal commitment, self-efficacy and regard for family factors were all associated with less alcohol consumption. |
| **Vossenaar M.** | 2008 | Participants respectively, from the rural and urban poor groups, from the urban middle class, with the highest degree of concordance with the WCRF/AICR guidelines were identified as PD. | Evaluation of specific eating habits (types of food) in PD in the three social classes (urban poor and urban middle class) | The FFQ for 14 individuals from each class were transformed into a day menu to create a rotating diet guide derived from members of each social group. |  | - |
| **Abildso C.** | 2021 | Counties classified into “high physical activity” (HPA) (PD) or “low physical activity” (LPA) (non-PD) based on county-level PA guideline prevalence for | Intercept interviews conducted with county coordinators and extension agents and other stakeholders, on-site observations |  |  | HPAs (PD) had (1) a culture/social norm that adults should engage in a greater variety of outdoor, lifetime types of PA; (2) a greater quantity and quality of built capital in place to support the multitude of activities (3) a greater quantity of human and organizational capital, with a longer track record of focus on PA |
| **Kraschnewski J.** | 2013 | PD were PCPs who performed weight counseling (identified through the National Ambulatory Medical Care Survey) | Logistic regression was performed to describe the association between visits to PD and non-PD physicians and receipt of weight counseling after adjusting for patient characteristics. |  |  | -52% of all weight counseling was done by 8.9% of PCPs, who performed weight counseling during at least 20% of visits.  -Adjusting for patient characteristics strengthened the relationship between a PD physician visit and provision of weight counseling. |
| **Curry L.** | 2011 | PD and non-PD were hospitals that ranked in either the top and the bottom 5% of performance respectively (measured by 30-day risk-standardized mortality rates due to acute myocardial infarction). | In-depth interviews conducted with key hospital staff who were most involved with AMI care in both low performing (non-PD) and high performing hospitals (PD) |  |  | In PD hospitals: 1) shared organizational values of providing exceptional, high-quality care (not apparent in non-PD hospital) 2) senior management and governing board more committed to high quality care and provide more financial and non-financial resources 3) presence of physician champions and empowered nursing staff, pharmacist involvement in patient care, and high qualification standards for all staff (all weak in non-PD hospitals) 4) Strong communication and coordination and better information flow in PD hospitals (all constrained in PD hospitals) |
| **David C.** | 2014 | Positive deviants (PD) were defined as individuals who had high diet quality at low diet cost. PD were identified using three separate measures of diet quality: Healthy Eating Index-2005 (HEI), Mean adequacy ratio (MAR), and Energy density (ED). HEI, MAR and ED. |  |  |  | -Individuals with high diet quality had similar HEI component scores.  -PD were able to achieve the same high HEI at lower cost. |
